# Where Should We Inject Botulinum Toxin for the Bunny Lines?

**DOI:** 10.1111/jocd.70673

**Published:** 2026-01-22

**Authors:** Kyu‐Ho Yi, Soo‐Bin Kim

**Affiliations:** ^1^ Division in Anatomy and Developmental Biology, Department of Oral Biology, Human Identification Research Institute, BK21 FOUR Project Yonsei University College of Dentistry Seoul Korea; ^2^ Department of Oral Anatomy, College of Dentistry Wonkwang University Iksan Korea

## 1 Introduction

**FIGURE 1:**
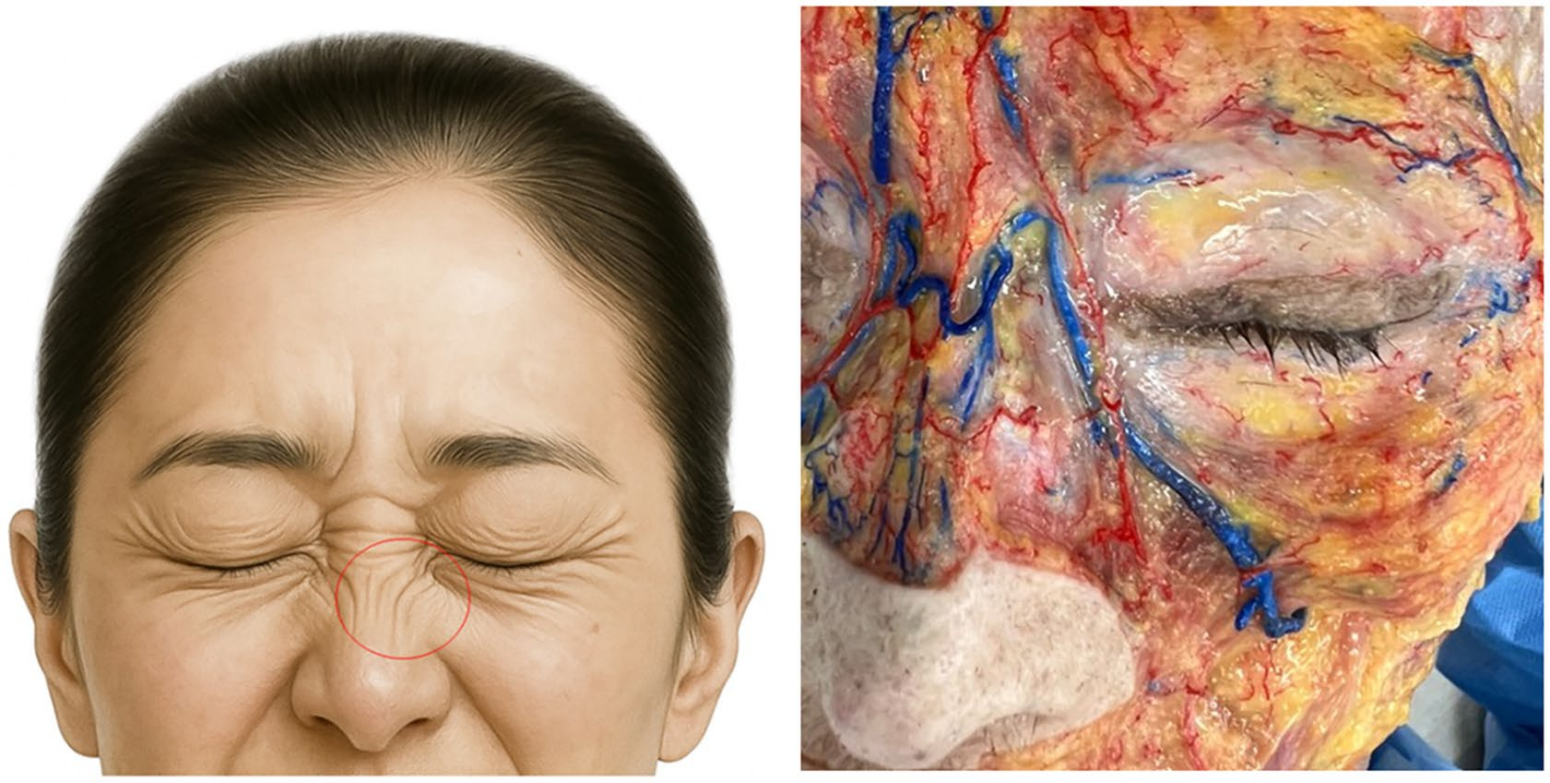
Bunny lines are fine, oblique wrinkles that appear on the upper lateral nasal bridge when smiling or squinting. They result from contraction of the transverse part of the nasalis muscle, sometimes assisted by the levator labii superioris alaeque nasi.

In aesthetic clinical practice, the treatment of “bunny lines”—the fine diagonal or vertical wrinkles that appear on the nasal sidewall during facial expressions such as smiling or laughing—has conventionally involved direct botulinum toxin (BoNT) injection into the crease itself (Figure 1). This approach assumes the presence of a hyperactive muscle immediately beneath the visible wrinkle. However, emerging evidence from detailed cadaveric dissections and ultrasonographic (US) analyses challenges this long‐standing assumption and may explain the inconsistent and sometimes suboptimal outcomes associated with the “in‐the‐line” injection approach [1].

Recent anatomical and imaging data demonstrate that the region between the medial canthus and nasal dorsum—where bunny lines manifest—is not underlain by a discrete muscle but rather represents a non‐muscular interval termed the bunny triangle. In their multimodal study involving 39 cadaveric dissections and dynamic US examinations of 13 volunteers, Ahn et al. [1] delineated this muscle‐free triangle bordered by the procerus (superior‐medial margin), transverse nasalis (superior‐lateral margin), levator labii superioris alaeque nasi (LLSAN, inferomedial margin), and medial fibers of the orbicularis oculi (OOc, lateral margin). During active nose scrunching, US confirmed that the visible wrinkles correspond to skin folding over this non‐muscular region, while adjacent muscles contract peripherally. The nasalis thickened dynamically in most participants, confirming its primary mechanical role in bunny line formation. Thus, the wrinkle is not generated by a localized underlying muscle but by convergent traction from neighboring muscles. Injecting BoNT directly into the crease therefore targets connective tissue and relies on passive diffusion, resulting in unpredictable efficacy and a higher likelihood of over‐ or under‐treatment depending on the diffusion pattern [2, 3].

The clinical implication of these findings is that the traditional “in‐the‐line” injection paradigm may be anatomically unsound. Instead, practitioners should adopt a border‐targeted chemodenervation strategy focusing on the muscles actually responsible for wrinkle formation. This concept aligns with modern principles of functional anatomy, emphasizing selective modulation of active muscle borders rather than superficial crease injection.

**FIGURE 2:**
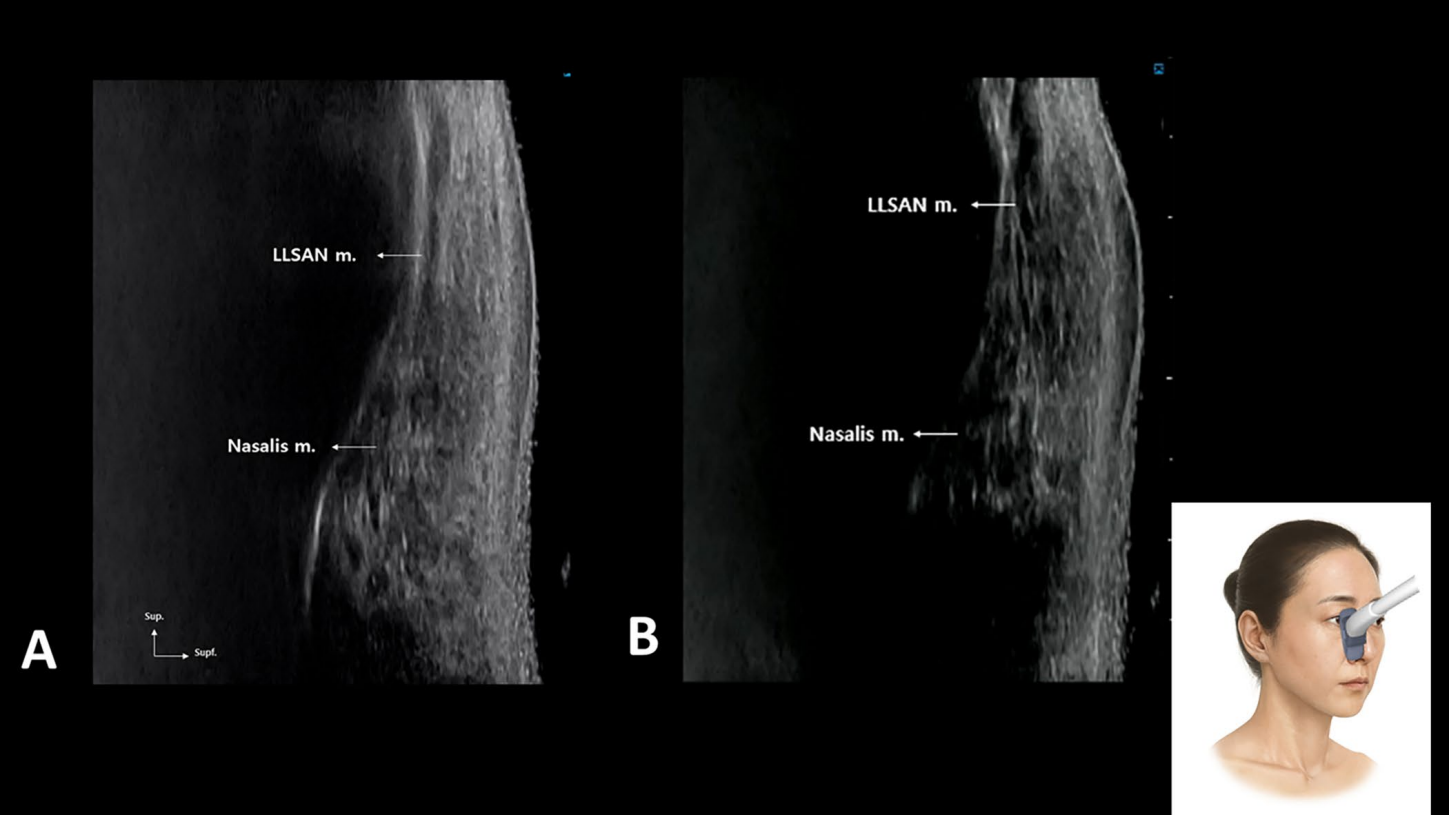
Resting (A) and contraction (B) of the levator labii superioris alaeque nasi and transverse part of the nasalis muscle.

Before injection, dynamic facial assessment should be performed. The patient is asked to smile, scrunch the nose, or express disgust to identify which borders contract most prominently—procerus, nasalis, LLSAN, or medial OOc. This individualized mapping acknowledges inter‐patient variability in perinasal muscle dominance (Figure 2).

## 2 Targeting the Borders Rather Than the Crease

**FIGURE 3:**
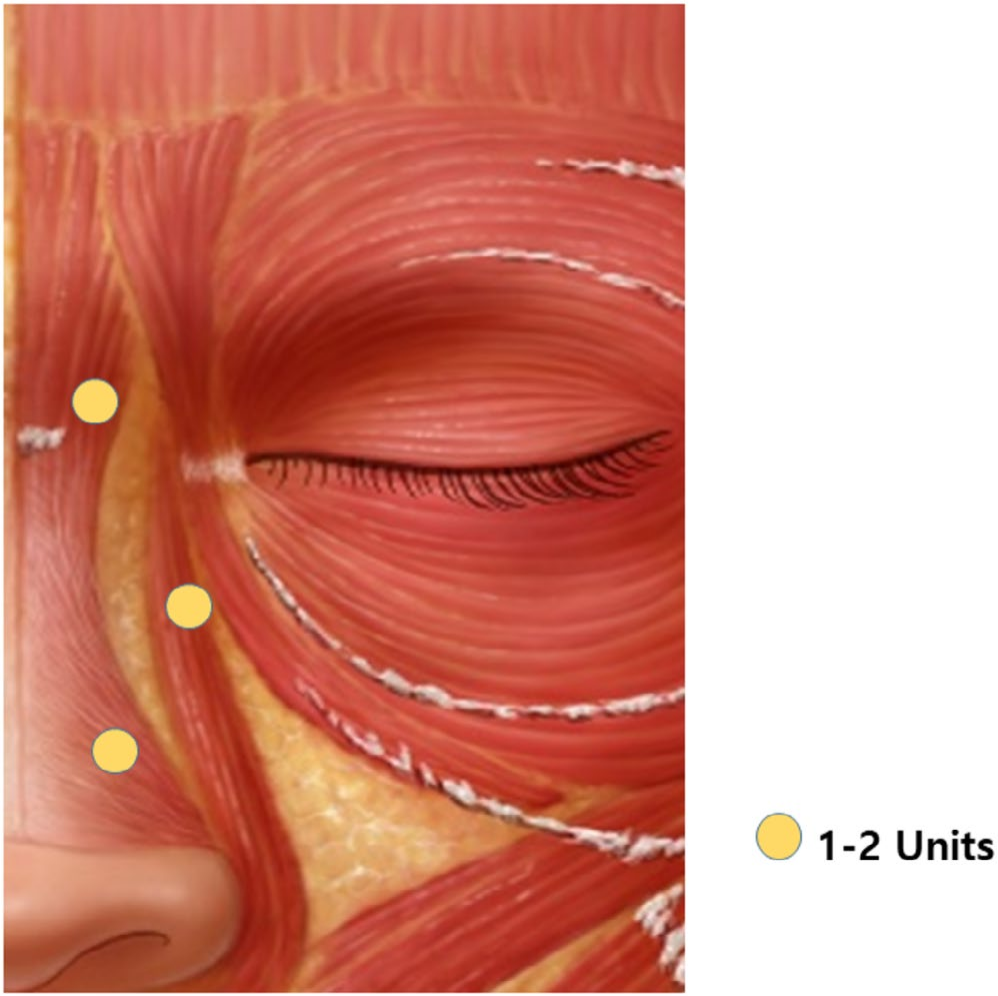
Targeting the borders rather than the crease. A dose of 1–2 units of JETEMA THE TOXIN (JETEMA Co. Ltd., Korea) was used per injection point (yellow dot).

(Figure 3) Transverse nasalis (primary driver): Small intramuscular injections (1–2 U of BoNT‐A) along its superior border on the upper nasal sidewall effectively attenuate line formation [4].

LLSAN (secondary contributor): Micro‐aliquots (0.5–1 U) at its superior‐medial border can suppress over‐elevation of the nasal ala but must be placed cautiously to avoid alar ptosis or upper‐lip elevator weakness.

Medial orbicularis oculi: When strong lateral traction accentuates the crease, a conservative injection at the medial canthal border is indicated.

Procerus: If glabellar contraction extends into the nasal root, a low‐dose (1 U) injection at its inferolateral margin may complement treatment.

High‐precision, low‐volume injections minimize toxin spread. Diffusion studies show that large volumes and low concentrations increase unintended denervation of neighboring muscles [5, 6]. Micro‐aliquots confined to active borders therefore improve both safety and predictability.

B‐mode US imaging allows real‐time visualization of the bunny triangle and contracting borders. US‐guided injection enhances accuracy, ensures correct depth (typically 1.5–2.0 mm in the subcutaneous plane), and avoids nearby vessels such as the angular artery. This technique also reduces the risk of asymmetry and diffusion‐related complications [7].

## 3 Mechanistic Rationale

Functionally, the bunny triangle acts as a passive deformation zone, folding when tension from surrounding muscles converges. The procerus provides superior medial pull, the transverse nasalis compresses laterally, and the LLSAN and medial OOc exert vertical and oblique traction, respectively [3]. Repeated recruitment of this synergistic complex induces dynamic wrinkles that later become static lines due to dermal thinning and repetitive traction [8]. Therefore, targeting the crease itself cannot effectively deactivate the muscular forces that generate it. Instead, injections at the periphery interrupt the contractile forces directly responsible for the fold.

This refined understanding also clarifies why excessive BoNT doses injected “into the line” can diffuse unpredictably to nearby elevators, leading to functional and aesthetic complications such as lip asymmetry, nasal alar descent, or lower‐eyelid weakness. Furthermore, repeated high‐dose injections in non‐muscular regions may increase the risk of antibody development, diminishing long‐term BoNT responsiveness [2].

## 4 Future Directions

While Ahn et al. [1] provided the first combined cadaveric–imaging correlation for the bunny triangle, additional prospective, controlled clinical studies are warranted. Direct comparisons between “in‐the‐line” and border‐targeted paradigms could validate improvements in wrinkle severity scores, patient satisfaction, and treatment longevity. Moreover, multimodal imaging combining US with surface electromyography could map contraction patterns in diverse ethnic populations, recognizing that perinasal muscular morphology varies by race and sex [4]. Establishing standardized injection coordinates relative to anatomical landmarks would further optimize reproducibility for both novice and experienced injectors.

## 5 Conclusions

Bunny lines do not correspond to an underlying muscle but occur over a non‐muscular zone formed by the convergence of adjacent facial‐expression muscles. The nasalis, LLSAN, medial orbicularis oculi, and procerus together produce these wrinkles by peripherally pulling the skin of the nasal sidewall. Therefore, effective and safe treatment requires targeting these muscle borders—not injecting into the wrinkle itself. We advocate for the adoption of ultrasound‐assisted, border‐specific BoNT injection protocols that reflect the true functional anatomy of the bunny triangle. By transitioning from empiric “in‐the‐line” injections to anatomically informed precision therapy, clinicians can achieve more predictable, natural, and enduring results.

## Author Contributions

Conceptualization, Writing – Original Draft, Visualization: Soo‐Bin Kim, Kyu‐Ho Yi. Writing – Review and Editing: Soo‐Bin Kim, Kyu‐Ho Yi. Supervision: Kyu‐Ho Yi.

## Funding

The authors have nothing to report.

## Consent

The authors have nothing to report.

## Conflicts of Interest

The authors declare no conflicts of interest.

## Data Availability

The data that support the findings of this study are available from the corresponding author upon reasonable request.
